# Synergistic Steatosis Induction in Mice: Exploring the Interactions and Underlying Mechanisms between PFOA and Tributyltin

**DOI:** 10.3390/cells13110940

**Published:** 2024-05-30

**Authors:** Yannick Dauwe, Lucile Mary, Fabiana Oliviero, Louise Dubois, Elodie Rousseau-Bacquie, Jelskey Gomez, Véronique Gayrard, Laïla Mselli-Lakhal

**Affiliations:** Toxalim (Research Centre in Food Toxicology), Université de Toulouse, Institut National de Recherche Pour L’agriculture, L’alimentation et L’environnement (INRAE), Ecole Nationale Veterinaire de Toulouse (ENVT), INP-Purpan, Université Paul Sabatier (UPS), 31027 Toulouse, France; yannick.dauwe@inrae.fr (Y.D.); lucileminie@hotmail.fr (L.M.); fabiana.oliviero@hotmail.it (F.O.); louise.dubois@inrae.fr (L.D.); elodie.rousseau-bacquie@inrae.fr (E.R.-B.); jelskey.gomes@inrae.fr (J.G.); veronique.gayrard@envt.fr (V.G.)

**Keywords:** nuclear receptor, PFAS, NAFLD, TBT, metabolism, synergism

## Abstract

This study explores the impact of environmental pollutants on nuclear receptors (CAR, PXR, PPARα, PPARγ, FXR, and LXR) and their heterodimerization partner, the Retinoid X Receptor (RXR). Such interaction may contribute to the onset of non-alcoholic fatty liver disease (NAFLD), which is initially characterized by steatosis and potentially progresses to steatohepatitis and fibrosis. Epidemiological studies have linked NAFLD occurrence to the exposure to environmental contaminants like PFAS. This study aims to assess the simultaneous activation of nuclear receptors via perfluorooctanoic acid (PFOA) and RXR coactivation via Tributyltin (TBT), examining their combined effects on steatogenic mechanisms. Mice were exposed to PFOA (10 mg/kg/day), TBT (5 mg/kg/day) or a combination of them for three days. Mechanisms underlying hepatic steatosis were explored by measuring nuclear receptor target gene and lipid metabolism key gene expressions, by quantifying plasma lipids and hepatic damage markers. This study elucidated the involvement of the Liver X Receptor (LXR) in the combined effect on steatosis and highlighted the permissive nature of the LXR/RXR heterodimer. Antagonistic effects of TBT on the PFOA-induced activation of the Pregnane X Receptor (PXR) and Peroxisome Proliferator-Activated Receptor Gamma (PPARγ) were also observed. Overall, this study revealed complex interactions between PFOA and TBT, shedding light on their combined impact on liver health.

## 1. Introduction

Presently, non-alcoholic fatty liver disease (NAFLD) is the principal cause of liver disease in the world. The worldwide prevalence of NAFLD is estimated to be at 34.4% [1]. It can induce various liver anomalies, starting from benign fat accumulation, known as steatosis, to inflammation with or without fibrosis, referred to as NASH, which can change into cirrhosis and hepatocellular carcinoma [2]. In total, 16% of NAFLD patients have NASH, and the global prevalence of NASH is estimated to be at 5.27% [3]. Among the global type 2 diabetes population, the prevalence of NAFLD is estimated to be 55.5%, in which the prevalence of NASH is 37.2% [4]. The prevalence of fibrosis affects 17% of the population with type 2 diabetes with NAFLD [4]. Obesity and dyslipidaemia are factors known to be associated with the majority of NAFLDs [5]. Nevertheless, these metabolic risk factors are not present in all cases of NAFLD. Other factors can contribute to this pathology, such as hepatitis C virus, prescription drugs, coeliac disease, genetic affections, and pollutants from the environment [6].

Exposure to various chemical groups of environmental contaminants have been correlated to NAFLD [7,8]. The epidemiological and mechanistic data have led to the concept of Toxicant-Associated Fatty Liver Disease (TAFLD) [9,10]. TAFLD implies that environmental contaminants can induce steatosis and contribute to the progression into more serious stages. Perfluorinated alkyls (PFAs) and Tributyltin (TBT) are known to have steatogenic properties [11,12,13]. Both PFOA and TBT induce steatosis by disrupting lipid metabolism in the liver [14,15,16,17]. These two compounds have been banned, but due to their persistence [18], humans continue to be exposed to them.

Environmental contaminants are known to interact with nuclear receptors involved in TAFLD [19,20]. Mechanisms contributing to steatogenesis, such as the induction of de novo lipogenesis, decreased fatty acid oxidation, increased import of free fatty acids into the liver, and decreased gluconeogenesis are known to be mediated by nuclear receptors [19]. Nuclear receptors, through their potential interaction with various chemicals, are suspected to play a crucial role in the cocktail effect. Recent research has therefore revealed that various chemicals can cooperatively bind to the ligand-binding pocket of the Pregnane X Receptor (PXR), leading to the synergistic activation of the receptor [21,22]. Moreover, these studies propose that a combination of environmental ligands of PXR and Retinoid X Receptor (RXR) can collaboratively initiate the recruitment of the steroid receptor coactivator-1 (SRC-1) by the heterodimer. This collaborative recruitment, in turn, was shown to lead to the synergistic activation of the PXR–RXR heterodimer and it’s target gene expression [22]. We also recently reported that the simultaneous exposure of mice to certain pesticides ligands of the Constitutive Androstane Receptor (CAR) combined with an RXR ligand can induce the synergistic activation of this nuclear receptor, also eliciting combined effects on steatosis and hypercholesterolemia, mainly through increased free fatty acid uptake and increased cholesterol synthesis [23].

The objective of this study was to investigate, in vivo, the potential coactivation of nuclear receptor heterodimers resulting from the co-exposure to an RXR ligand with another environmental contaminant group, namely the PFAS group, which is known to interact with multiple nuclear receptors [24,25,26]. We selected tributyltin (TBT), a well-known pollutant acting as an RXR ligand [27], and perfluorooctanoic acid (PFOA), as the representative of the PFAS group. The primary focus was placed on studying the synergic activation of CAR, PXR, Peroxisome Proliferator-Activated Receptor Alpha (PPARα), Peroxisome Proliferator-Activated Receptor Gamma (PPARγ), Liver X Receptor (LXR), and Farnesoid X Receptor (FXR) through the upregulation of their prototypical target genes. As a second step, we assessed whether a combined effect could be observed on lipid metabolism disruption and steatosis, with the ultimate goal being to provide a mechanistic insight into the observed effects.

## 2. Materials and Methods

### 2.1. Animal Experiment

This in vivo study adhered to the guidelines outlined by the European Union for the use and care of laboratory animals. An independent ethics committee (Toxcométhique, INRAE Toxalim, Toulouse, France) granted approval for the experiment (Approval Code: APAFIS#21271-2019062816356401). Nine-week-old male C57BL/6 J mice sourced from the Janvier Lab (Le Genest-Saint-Isle, France) were utilized for this experiment. They were housed in Type S polycarbonate cages (Charles River, Ecully, France) under controlled conditions: temperature ranging from 20 °C to 24 °C, with a 12 h light/dark cycle, and provided with ad libitum access to food and water. The housing environment was enriched with a stainless-steel hut to offer shelter and reduce stress. The mice were divided into four groups, each consisting of six individuals, and were force-fed once daily for a duration of 3 days. We used dimethyl sulfoxide (DMSO ref. 276855, Saint-Quentin Fallavier, France) for the control group, with the interventions comprising TBT (5 mg/kg/day ref. T50202, Saint-Quentin Fallavier, France), PFOA (10 mg/kg/day ref. 171468, Saint-Quentin Fallavier, France), and TBT + PFOA (a combination of both). 

After 3 days, each mouse’s body weight was measured. Then, a blood sample was taken from the submandibular vein using a lancet and placed in an EDTA-coated tube (BDMicrotainer^®^; BD, Le Pont-de-Claix, France). Plasma was obtained via centrifugation (1500× *g* for 10 min at 4 °C) and stored at −80 °C for biochemical analyses. Then, the animals were euthanized via cervical dislocation. The mice’s livers where collected, weighed, snap-frozen in liquid nitrogen and stored at −80 °C for further use.

### 2.2. Histology

The cryocuts and staining were performed on the liver samples as previously described by [23]. The area covered by lipid droplets was assessed using the public domain software ImageJ V1.54i (ImageJ website: https://imagej.net/ij/, accessed on 1 August 2023). Steatosis was defined as lipid vesicle coverage of 5% or more. Lipid droplet count and average size was determined using the particle analysis function of ImageJ.

### 2.3. Liver Neutral Lipid Analysis

The hepatic neutral lipid contents were determined as previously described in [28]. Briefly, the liver samples were homogenized in methanol/5 mM EGTA (2:1, *v*/*v*), followed by a lipid extraction with chloroform/methanol/water (2.5:2.5:2.1, *v*/*v*/*v*). Glyceryl trinonadecanoate, stigmasterolm, and cholesteryl heptadecanoate (Sigma) were included as internal standards. The triglycerides (TGs), free cholesterol, and cholesterol esters were analyzed via gas–liquid chromatography with a Focus Thermo Electron system from Thermo Scientific (Pittsburgh, PA, USA).

### 2.4. Plasma Analysis

Alanine aminotransferase (ALAT), aspartate amino transferase (ASAT), TG, free fatty acid (FFA), cholesterol, high-density lipoprotein (HDL), and low-density lipoprotein (LDL) levels were established using an ABX Pentra 400 biochemical analyzer (Horiba Medical, Anexplo facility, Toulouse, France).

### 2.5. Gene Expression Studies

RNA extraction, reverse transcription, RT-qPCR, and analysis were performed as outlined previously in [23]. The RT-qPCR primers are presented in Table 1.

### 2.6. Combinatorial Effects and Statistical Analysis

Gene expression levels were converted to fold changes relative to the DMSO-treated group. Statistical analyses were conducted using GraphPad Prism 10. Each dataset underwent analysis using a one-way ANOVA test followed by Tukey’s multiple comparisons test, with significance set at *p* < 0.05.

To assess combined effects, we employed different approaches as detailed in [29]: the “Combination Subthresholding approach” and the “highest single agent approach/cooperative effect”. In summary, the “Combination Subthresholding approach” consists of showing that a combination of noneffective doses of drugs yields a significant effect, and the “highest single agent approach/cooperative effect” reflects that the resulting effect of a drug combination is greater than the effects produced by its individual components [29]. Where applicable, we utilized the “response additivity” model, calculating the additivity as E_TBT+PFOA_ = E_TBT_ + E_PFOA_. Synergistic effects were identified when they significantly exceeded the additive effect, determined using a one-sample t-test. Finally, a potentiation effect was identified when the effect of compound A was increased by another compound (B), which did not induce any effects [30].

## 3. Results

### 3.1. Potentiation Effect on Steatosis Induction by Tributyltin and PFOA

This investigation assessed the impacts of individual and combined impacts of PFOA and TBT treatments on the body and liver weights of mice after sacrifice. There was no significant difference in body weight between the different groups. However, the liver/body weight ratio was found to be significantly higher in mice treated with PFOA and TBT + PFOA compared to DMSO-treated mice (control) (Table 2). The combined treatment (TBT + PFOA) did not induce a significantly higher ratio than PFOA alone.

**Figure 1:**
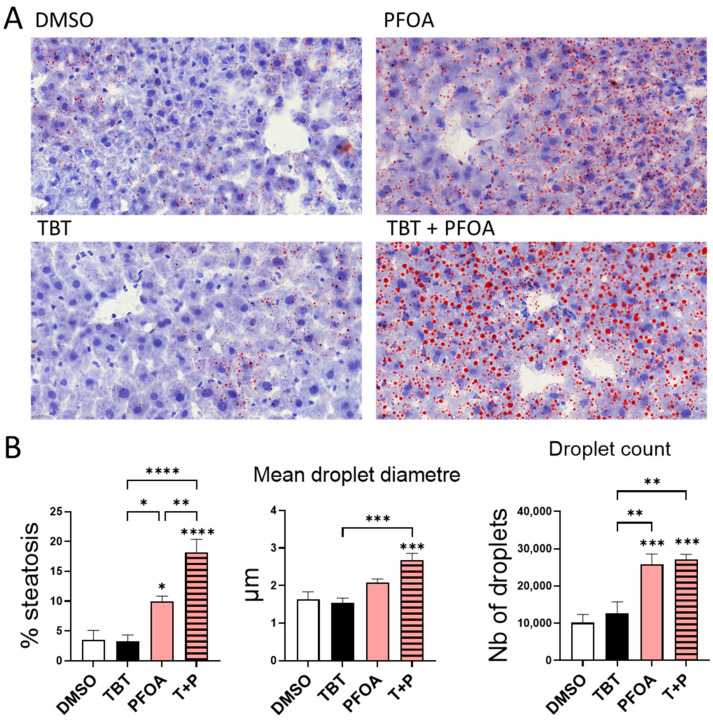
Histologic hepatic steatosis assessment. Histology was conducted on frozen liver sections stained with Harris Haematoxylin and red oil stain. (**A**) An accumulation of multiple small red droplets in the cytoplasm allows to assess steatosis. Zoom focus: 63×. (**B**) Optic quantification and morphological assessment of steatosis. Area of lipid droplets and droplet diameters are presented as mean ± standard error of the mean. The *p*-values indicate the level of significance, with * *p* < 0.05, ** *p* < 0.01, *** *p* < 0.001 and **** *p* < 0.0001 being calculated via the one-way ANOVA test followed by Tukey’s multiple comparisons test.

Histological sections of the liver underwent staining with red oil to examine lipid accumulation (Figure 1). TBT did not induce steatosis, while PFOA did exhibit this effect. More interestingly, the combined treatment induced a higher percentage of steatosis compared to PFOA treatment alone, indicating a potentiation effect of TBT on PFOA. For PFOA alone and combined with TBT, the lipid droplets were microvesicular and mostly followed an azonal distribution. When TBT was added to PFOA, the average lipid droplet size increased, while the average count remained stable. This suggests that TBT potentiates PFOA-induced steatosis by increasing the lipid content of lipid droplets through swelling, without increasing additional droplet production.

### 3.2. Combined Effects of Tributyltin and PFOA on Lipid Accumulation in the Liver

Hepatic neutral lipid levels were examined to determine the quantity of previously characterized steatoses (Table 3). The primary constituents of lipid droplets were examined, and triglycerides (TGs), cholesteryl esters, and cholesterol were quantified. A subthreshold effect occurred with TBT + PFOA on TG accumulation (a fold change of 3.64). These results are in agreement with the combined effect on steatosis, in accordance with the histological analysis (Figure 1). It is noteworthy that the observed combined effects cannot be defined as synergistic but rather additive because they were not significantly higher than the calculated additive effect. The results showed no effect on cholesterol levels. Cholesterol ester levels where not significantly changed by the individual components but decreased when the combined treatment was used.

### 3.3. Changes in Plasmatic Biochemical Profiles

Liver function markers were evaluated within each group, including ALAT, ASAT, TG, free fatty acid (FFA), total cholesterol, HDL, and LDL levels (Table 4). The plasma levels of ALAT, the most used marker of liver injury, were increased by the mixture but not by the components alone, suggesting a combined subthreshold effect on liver damage linked to TAFLD. PFOA alone decreased the TG levels by 42% and by 47% when it was combined with TBT. No additive effect of TBT was observed when combined with PFOA. LDL levels were increased by TBT alone, but this increase was cancelled out when TBT was combined with PFOA. ASAT, FFA, cholesterol, and HDL levels remained stable, regardless of treatment.

### 3.4. Multiple Nuclear Receptor Modulations

**Figure 2:**
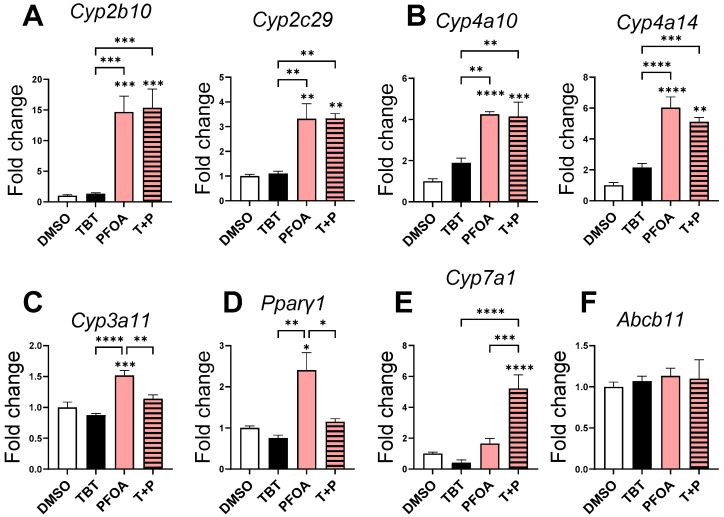
Nuclear receptor activation in the liver. RT-qPCR analysis was performed on (**A**) CAR prototypical target genes (*Cyp2b10* and *Cyp2c29*); (**B**) PPARα target genes *Cyp4a10* and *Cyp4a14*; (**C**) PXR prototypical target gene *Cyp3a11*; (**D**) *Pparγ* expression; (**E**) LXR target gene *Cyp7a1*; (**F**) FXR target gene *Abcb11*. The results are presented as a graph, showing the expression levels in fold changes of the DMSO group. The data are presented as mean ± standard error of the means. The statistical analysis used was a one-way ANOVA test followed by Tukey’s multiple comparisons test. The *p*-values indicate the level of significance, with * *p* < 0.05, ** *p* < 0.01, *** *p* < 0.001, and **** *p* < 0.0001 indicating significant differences.

To determine which nuclear receptors could be involved in the steatogenic effects of PFOA and the potentiating effect of TBT, we evaluated the coactivation of CAR-RXR, PPARα-RXR, PXR-RXR, PPARγ-RXR, LXR-RXR, and FXR-RXR heterodimers with PFOA and TBT by measuring the expression of prototypical target genes of each receptor (Figure 2). The activation of the CAR-RXR heterodimer was measured by analyzing the expression of the CAR target genes *Cyp2b10* and *Cyp2c29*. No significant activation was observed for TBT alone. PFOA treatment alone or combined with TBT activated CAR in the same manner (Figure 2A). The same activation profiles were observed for PPARα target genes *Cyp4a10* and *Cyp4a14* (Figure 2B). PXR activation was assessed through the expression of its prototypical target gene *Cyp3a11* (Figure 2C). PFOA alone activated PXR, but the combination with TBT suppressed this activation, indicating an antagonistic effect. A similar effect occurred on PPARγ, whose expression is considered as a marker of its own activation (Figure 2D). LXR activation was assessed through *Cyp7a1* expression levels. LXR was not activated by PFOA or TBT alone but by the mixture of the two components, evidencing a subthreshold effect (Figure 2E). FXR activity, assessed through its target gene *Abcb11*, was not altered by these treatments (Figure 2F).

### 3.5. The Cocktail Effect of PFOA and TBT on Genes Involved in Steatogenesis in the Liver

To assess the possible mechanisms related to the combined effect on steatosis induced by just PFOA or in combination with TBT (Figure 1, Table 3), we evaluated the expression of essential genes implicated in distinct hepatic metabolic pathways, such as lipogenesis, lipid droplet formation, fatty acid β-oxidation, lipid transport, and cholesterol ester import (Table 5).

**Figure 3:**
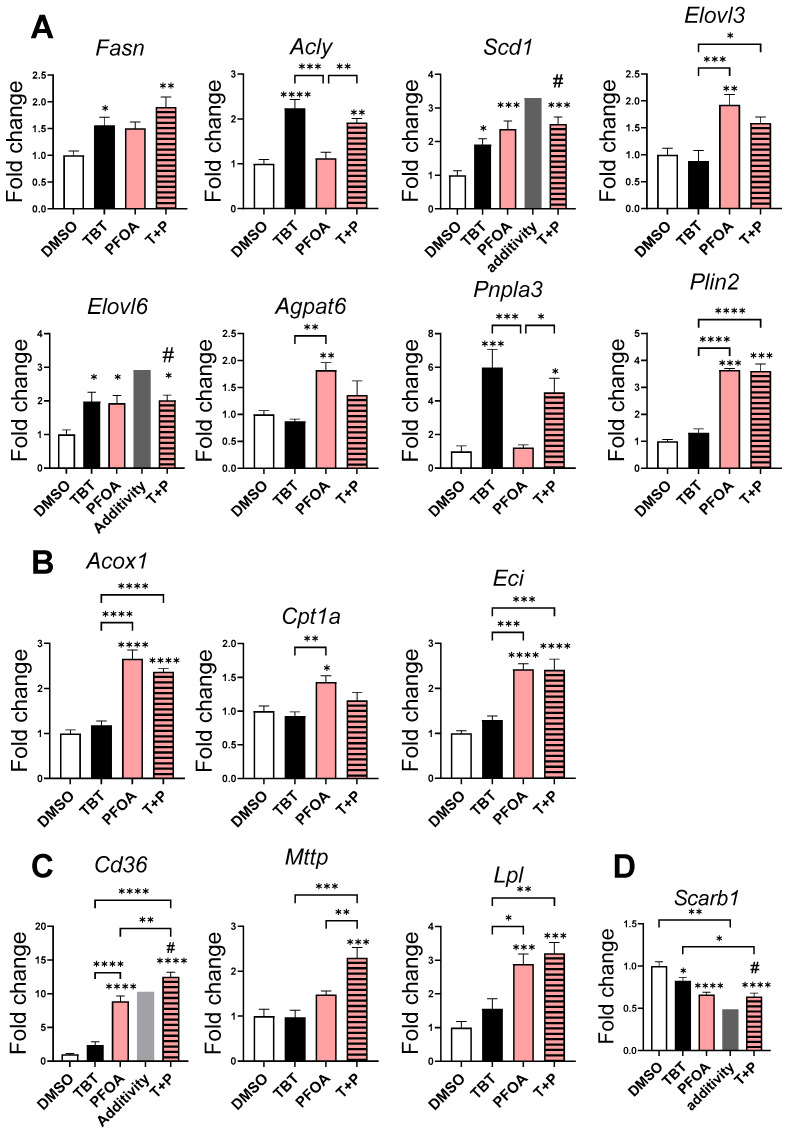
RT-qPCR of genes involved in lipid metabolism and transport. RT-qPCR of genes involved in (**A**) lipid synthesis and lipid droplet formation, (**B**) in fatty acid β-oxidation, (**C**) in fatty acid and triglyceride transport, and (**D**) in cholesteryl ester uptake. The results are expressed as fold changes of the DMSO group. Data are presented as mean ± standard error of the mean. * *p* < 0.05, ** *p* < 0.01, *** *p* < 0.001, **** *p* < 0.0001. *p*-values represent significant differences between each treatment group according to one-way ANOVA test followed by Tukey’s multiple comparisons test. # for *p* < 0.05 represents a significant difference, with additivity calculated using a one-sample t-test.

PFOA induced an upregulation of genes involved in lipogenesis, particularly during desaturation (such as *Scd1*), in elongation (such as *Elovl3*, and *Elovl6*), in lipid droplet formation (such as *Plin2*), and in phospholipid biosynthesis (like *Agpat6*) (Figure 3A). It also upregulated genes involved in lipid catabolism (such as *Cpt1a*, *Acox1*, and *Eci*) (Figure 3B) as well as in fatty acid uptake (like *Cd36*) and release (such as *Lpl*) (Figure 3C).

TBT induced the upregulation of genes involved in the first steps of lipogenesis (such as *Fasn* and *Acly*) but also in elongation (such as *Elovl6*) and desaturation (such as *Scd1* and *Pnpla3*), which influences the balance between lipid storage and mobilization in liver cells (Figure 3A).

Regarding the interaction of the two molecules, three types of combined effects could be distinguished. The first one consisted of positive interactions between compounds—such as a synergistic upregulation of *Cd36* and a combined subthreshold effect on *Mttp*, which are involved in triglyceride-rich lipoprotein formation, particularly very low-density lipoprotein (VLDL) (Figure 3C). For the second type of combined effect, the same effect levels were observed with TBT, PFOA, and PFOA + TBT. No additive or synergistic effect was therefore evidenced when the results were compared to the calculated additivity for *Elovl6* and *Scd1* (Figure 3A). Simultaneously, the expression of *Scarb1* was downregulated by TBT and PFOA. However, the combined effect of TBT + PFOA was lower than additivity (Figure 3D). The last type was characterized by an antagonist effect of TBT on the upregulation of *Elovl3*, *Agpat6*, and *Cpt1a* by PFOA, which completely cancelled out the effect.

## 4. Discussion

This study aimed to assess the combined effects of PFOA and TBT on the induction of steatosis, the activation of nuclear receptors, and the expression of genes involved in hepatic lipid metabolism. This study highlighted different types of effects when PFOA and TBT were associated. These effects included both positive interactions on steatosis and the expression of certain genes, as well as the specific effects and antagonistic effects of PFOA and TBT. The investigation also provided insights into the molecular mechanisms underlying the combined effects on the decreased cholesterol ester levels and increased triglyceride levels.

The steatogenic effects of PFOA and TBT have been individually investigated in other studies [11,13,16,17]. The originality of this study lies in its evaluation of how these compounds interact to induce combined effects on steatosis. In our conditions, TBT did not induce steatosis when administered alone, but it exacerbated the steatosis induced by PFOA (Figure 1 and Table 3). This study also found that the combined effect on steatosis led to increased hepatic suffering, as evidenced by the higher ALAT levels, pointing in the direction of a faster progression into more serious states of Toxicant-Associated Fatty Liver Disease (TAFLD) (Table 4).

PFOA induced the activation of several nuclear receptors and the expression of key genes involved in various hepatic metabolic pathways. It triggered both the activation of genes involved in lipogenesis and beta oxidation, which represents a paradox where conflicting pathways of synthesis and catabolism are simultaneously activated (Figure 3A,B). However, the modulation of expression regarding these genes remained low, namely a 2.5 times higher expression at most, compared to that of the *Cd36* transporter involved in the uptake of circulating fatty acids, which exhibited a 12.5-time higher expression. These results suggest that the import of fatty acids through *Cd36* and the activation of lipogenesis outweigh fatty acid catabolism, leading to the observed lipid accumulation (Figure 1 and Table 3). The hypothesis of increased lipid importation is supported by the decreased plasma triglyceride levels (Table 4) and by other data, which show that PFOA induces lipid accumulation in the liver [11,31] and affects hepatic lipid metabolism by disrupting fatty acid trafficking [32]. In our study, we did not replicate the decrease in plasma free fatty acids, which was evidenced by another prior study [31]. This difference could be attributed to a longer exposure period of 7 days, in comparison to the 3 days utilized in our study. Increased triglyceride levels as well as altered lipid and cholesterol metabolism genes in mice livers have also been seen in human cells [33,34]. These studies have suggested an involvement of the PPARα receptor [33], as well as the Hepatocyte Nuclear Factor 4 alpha (HNF4α) transcription factor [34], in the steatogenic effect of PFOA. The PPARα receptor is generally considered to be the primary target of PFOA [35,36,37]. In our study, we showed that in addition to activating PPARα, PFOA also activates CAR, PXR, and PPARγ. A distinctive characteristic of CAR is its constitutive activity, implying its ability to initiate the expression of target genes even in the absence of ligand binding [38]. The activation of CAR, triggered by a xenobiotic, leads to its detachment from the cytoplasmic retention complex, enabling its translocation to the nucleus. In the nucleus, CAR can then stimulates the expression of its prototypical target genes [39]. While PFOA is recognized as a CAR activator, it does not directly act as an agonist [25]. Instead, PFOA indirectly activates CAR, but it does not carry out this process exclusively through the epithelial growth factor (EGFR) coupled with the protein phosphatase 2A (PP2A) dephosphorylation pathway [24]. Despite examining indirect CAR activation and direct RXR activation in this study, a synergistic effect on CAR/RXR was not corroborated with the agonistic pesticides of CAR combined with TBT (as it was in recently published data) [23].

Interestingly, this study rules out the involvement of the farnesoid X receptor (FXR), as no specific modulations in the FXR target gene *Abcb11* were observed. The study suggested that the Liver X Receptor (LXR) was the primary factor contributing significantly to the combined effect on steatosis, since synergistic effects were evidenced only on LXR target genes, namely *Cyp7a1* and *Cd36* [40]. The activation of LXR by xenobiotics has been previously reported to lead to the development of hepatic steatosis [41] through *Cd36* upregulation [40]. Another interesting point raised by our study concerns the permissive nature of the LXR/RXR heterodimer. Previous studies have identified two distinct categories of nuclear receptor heterodimers: permissive and nonpermissive [42]. Heterodimers classified as permissive can be activated by ligands binding to either RXR or its partner receptor, with their activation being synergistically heightened in the presence of both ligands [43]. On the contrary, nonpermissive heterodimers cannot be activated by ligands that bind specifically to RXR, resulting in RXR functioning as a “silent” partner in these instances [43]. However, the permissiveness or nonpermissiveness of a given heterodimer is contingent on various factors, such as specific ligands, DNA sequences, cellular environment, and post-translational modifications [44]. Our findings confirm the permissiveness of the LXR/RXR complex, showcasing that activation is facilitated by TBT. Indeed, while the TBT treatment did not activate any of the tested nuclear receptors, it activated LXR when combined with PFOA, likely trough RXR. Previous research using cell reporter assays for human, murine, and rat receptors indicates that PFOA does not directly activate LXR or RXR [45]. This suggests that PFOA’s observed LXR activation might be indirect, involving other mechanisms or endogenous ligands.

LXR was shown to exhibit both hypocholesterolemic characteristics by increasing hepatic catabolism through the upregulation of enzymes controlling bile acid synthesis, such as CYP7A1 [46]. However, it also has hypertriglyceridemic effects by activating genes involved in lipogenesis and fatty acid import, such as *Cd36*. The activation of LXR could therefore explain why the steatosis observed in our study was primarily composed of triglycerides with few cholesterol esters. Furthermore, both PFOA and TBT were found to decrease the expression of *Scarb1*, a gene responsible for cholesterol ester uptake through high-density lipoprotein (HDL) uptake. This gene is known to be downregulated by LXR activation [47]. The present study is an illustration of the adverse outcome pathways associated with steatosis, focusing on the pivotal role of nuclear receptor activation or inhibition as the initiating events [48]. Notably, our investigation uncovers the specific effects of perfluorooctanoic acid (PFOA) and highlights the intriguing, combined impact when PFOA is introduced in conjunction with TBT.

In their recent investigation, Delfosse et al. illustrated that the synergic activation of PXR may encompass not only the binding of multiple molecules to the ligand pocket of the receptor but also the concurrent activation of the two components of the PXR–RXR heterodimer [21,22]. This implies that environmental ligands for both PXR and RXR can collaboratively induce the recruitment of the coactivator steroid receptor coactivator-1 (SRC-1) by the heterodimer, leading to the synergistic activation of PXR–RXR target gene expression. What distinguishes our study from others is its confirmation that such synergistic effects of numerous environmental contaminants on nuclear receptors occur not solely in vitro but also in vivo, and that they occur with another nuclear receptor, LXR, in addition to PXR [22] and CAR [23].

Another type of interaction observed in our study is the antagonistic effect of TBT on PFOA-induced PXR and PPARγ activation (Figure 2C,D). The mechanisms involved in this antagonistic effect on a heterodimer with RXR have been relatively unexplored in the literature. It may likely involve the recruitment of co-repressors that prevail over coactivator recruitment [49]. It would be interesting to subsequently determine why the binding of TBT to RXR led to coactivator recruitment in the context of the LXR/RXR complex and to co-repressor recruitment in the cases of PXR/RXR and PPARγ/RXR complexes.

## 5. Conclusions

In summary, this study identified the intricate interactions between environmental contaminants, specifically PFOA and TBT, and their combined impact on TAFLD. This research uncovered a spectrum of effects, ranging from the synergistic to antagonistic activation of nuclear receptors, like PXR, LXR, and PPARγ. Notably, the combination of PFOA and TBT led to a potentiation effect on steatosis, with TBT intensifying the steatogenic properties of PFOA. We have highlighted a possible mechanism involving the activation of LXR and its target genes, including the fatty acid transporter *Cd36*, which could explain the potentiating effect of TBT on PFOA-induced steatosis.

## Figures and Tables

**Table 1:** Sequence of the primers used in RT-qPCR.

Gene	Primer Sequence F	Primer Sequence R
*Abcb11*	ACTTCTGTGGGAGAGCTCAATTC	GTCGGCAATGGCTTCATCAATTT
*Acly*	AAAGCTTGGCCTCGTCGG	GGGACGAAGGGTTCAATGAGA
*Acox1*	AGACCCTGAAGAAATCATGTGG	AGGAACATGCCCAAGTGAAG
*Agpat6*	CAGCTGTACAAGCCCTACACCA	AGCTTTACTACTACCACTTCGACGAAT
*Cd36*	GTTAAACAAAGAGGTCCTTACACATACAG	AGTGAAGGCTCAAAGATGGC
*Cpt1a*	GAAGAAGAAGTTCATCCGATTCAAG	GATATCACACCCACCACCACG
*Cyp2b10*	TTTCTGCCCTTCTCAACAGGAA	TGGACGTGAAGAAAAGGAACAAC
*Cyp2c29*	GCTCAAAGCCTACTGTCA	CATGAGTGTAAATCGTCTCA
*Cyp3a11*	TCACACACACAGTTGTAGGCAGAA	GTTTACGAGTCCCATATCGGTAGAG
*Cyp4a10*	ATTAGTGAGAGTGAGGACAGCAACAG	CCAACCCGATTTGCAGACA
*Cyp4a14*	TCAGTCTATTTCTGGTGCTGTTC	GAGCTCCTTGTCCTTCAGATGGT
*Cyp7a1*	AGCAACTAAACAACCTGCCAGTACTA	GCCGCAGAGCCTCCTTG
*Eci*	GTTCACCATCAGCCTGGAGAAG	AGAAGATACCCGGGCATTCC
*Elovl3*	GCCTCTCATCCTCTGGTCCT	TGCCATAAACTTCCACATCCT
*Elovl6*	TCTGATGAACAAGCGAGCCA	TGGTCATCAGAATGTACAGCATGT
*Fasn*	AGTCAGCTATGAAGCAATTGTGGA	CACCCAGACGCCAGTGTTC
*Lpl*	ATGGCAAGCAACACAACCAG	TGTGGAAACCTCGGGCAG
*Mttp*	TCAGGAAGCTGTGTCAGAATGAAG	TTTCAAGTCCTCCCAGGATCA
*Plin2*	CCATTTCTCAGCTCCACTCCAC	GTGTCGTCGTAGCCGATGC
*Pnpla3*	ACGCGGTCACCTTCGTGT	AGCCCGTCTCTGATGCACTT
*Pparg1*	GACCAACAGCCTGACGGG	TGAATATCAGTGGTTCACCGCTT
*Scarb1*	TCCCTCATCAAGCAGCAGGT	ACCTCGTTTGGGTTGACCAC
*Scd1*	CAGTGCCGCGCATCTCTAT	CAGCGGTACTCACTGGCAGA
*Tbp*	ACTTCGTGCAAGAAATGCTGAA	GCAGTTGTCCGTGGCTCTCT

**Table 2:** Body and liver weights. Body weight in grams and effects of treatments on liver/body weight ratio are expressed as the fold change of the control mice. The results are shown as mean ± standard error of the mean (SEM). “*” indicates significant difference with DMSO. *p* < 0.05 is considered significant according to one-way ANOVA test followed by Tukey’s multiple comparisons test.

Treatment	Body Weight (g)	Liver/Body Weight
DMSO	24.80 ± 0.48	1.00 ± 0.027
TBT	24.22 ± 0.45	1.08 ± 0.050
PFOA	24.43 ± 0.40	1.28 ± 0.028 *
TBT + PFOA	25.10 ± 0.54	1.39 ± 0.021 *

**Table 3:** Liver lipid quantification. Effects of PFOA and TBT treatments on hepatic levels of triglycerides, cholesteryl esters, and total cholesterol. Results are expressed in relative abundance for 1 mg of liver and fold change of DMSO group (mean ± standard error of the mean). “*” indicates significant difference with DMSO; “^T^” with TBT; “^P^” with PFOA. *p* < 0.05 is considered significant according to one-way ANOVA test followed by Tukey’s multiple comparisons test.

	Triglycerides		Cholesteryl Esters		Cholesterol	
Treatment	Abundance%	Fold Change	Abundance%	Fold Change	Abundance%	Fold Change
DMSO	3.3 ± 0.42	1.00 ± 0.128	0.078 ± 0.0069	1.00 ± 0.08	0.36 ± 0.024	1.00 ± 0.08
TBT	4.6 ± 0.97	1.39 ± 0.29	0.073 ± 0.0069	0.93 ± 0.08	0.34 ± 0.036	0.93 ± 0.10
PFOA	7.1 ± 0.62	2.17 ± 0.18	0.078 ± 0.0081	0.99 ± 0.10	0.37 ± 0.029	1.03 ± 0.08
TBT + PFOA	11.9 ± 1.99 *^TP^	3.64 ± 0.60 *^TP^	0.046 ± 0.0028 *^TP^	0.58 ± 0.03*^TP^	0.30 ± 0.02	0.84 ± 0.06

**Table 4:** Plasma biochemical parameters. Effects of PFOA and TBT treatments on plasmatic biochemical parameters. Results are expressed as fold change of DMSO group (mean ± standard error of the mean). “*” represents significant difference with DMSO. *p* < 0.05 is considered significant according to one-way ANOVA test followed by Tukey’s multiple comparisons test.

Treatment	TBT	PFOA	TBT + PFOA
ALAT	1.49 ± 0.218	2.09 ± 0.316	3.09 ± 0.576 *
ASAT	1.04 ± 0.042	1.37 ± 0.222	1.18 ± 0.099
TG	0.77 ± 0.075	0.58 ± 0.047 *	0.53 ± 0.013 *
FFA	1.49 ± 0.210	1.56 ± 0.226	1.19 ± 0.056
Cholesterol	1.00 ± 0.075	0.88 ± 0.038	0.82 ± 0.025
HDL	1.06 ± 0.087	0.98 ± 0.043	0.96 ± 0.025
LDL	1.36 ± 0.064 *	0.92 ± 0.106	0.77 ± 0.043

**Table 5:** Key genes involved in liver lipid metabolism.

Function	Gene	Protein
Lipogenesis	*Fasn*	Fatty acid synthase
	*Acly*	ATP Citrate Lyase
	*Scd1*	Stearoyl-CoA desaturase 1
	*Elovl3*	Fatty Acid Elongase 3
	*Elovl6*	Fatty Acid Elongase 6
	*Agpat6*	1-acylglycerol-3-phosphate O-acyltransferase 6
	*Pnpla3*	Patatin-like phospholipase domain containing 3
	*Plin2*	Perilipin 2
β-oxydation	*Acox1*	Peroxisomal acyl-coenzyme A oxidase 1
	*Cpt1a*	Carnitine palmitoyltransferase 1a
	*Eci*	Enoyl-CoA Delta Isomerase
Lipid transport	*Cd36*	Fatty acid translocase
	*Mttp*	Microsomal triglyceride transfer protein
	*Lpl*	Lipoprotein lipase
Cholesteryl ester import	*Scarb1*	Scavenger receptor class B member 1

## Data Availability

The data presented in this study are available upon request from the corresponding author.
